# Measurement of the plasma levels of antibodies against the polymorphic vaccine candidate apical membrane antigen 1 in a malaria-exposed population

**DOI:** 10.1186/1471-2334-12-32

**Published:** 2012-02-02

**Authors:** Kwadwo A Kusi, Daniel Dodoo, Samuel Bosomprah, Marjolein van der Eijk, Bart W Faber, Clemens HM Kocken, Edmond J Remarque

**Affiliations:** 1 Department of Parasitology, Biomedical Primate Research Centre, Postbox 3306, 2280, GH Rijswijk, The Netherlands; 2 Department of Immunology, Noguchi Memorial Institute for Medical Research, College of Health Sciences, University of Ghana, P.O. Box LG581, Legon, Accra, Ghana; 3 Department of Biostatistics, School of Public Health, University of Ghana, P. O. Box LG13, Legon, Accra, Ghana

## Abstract

**Background:**

Establishing antibody correlates of protection against malaria in human field studies and clinical trials requires, amongst others, an accurate estimation of antibody levels. For polymorphic antigens such as apical membrane antigen 1 (AMA1), this may be confounded by the occurrence of a large number of allelic variants in nature.

**Methods:**

To test this hypothesis, plasma antibody levels in an age-stratified cohort of naturally exposed children from a malaria-endemic area in Southern Ghana were determined by indirect ELISA. Titres against four single *Pf*AMA1 alleles were compared with those against three different allele mixtures presumed to have a wider repertoire of epitope specificities. Associations of antibody levels with the incidence of clinical malaria as well as with previous exposure to parasites were also examined.

**Results:**

Antibody titres against *Pf*AMA1 alleles generally increased with age/exposure while antibody specificity for *Pf*AMA1 variants decreased, implying that younger children (≤ 5 years) elicit a more strain-specific antibody response compared to older children. Antibody titre measurements against the FVO and 3D7 AMA1 alleles gave the best titre estimates as these varied least in pair-wise comparisons with titres against all *Pf*AMA1 allele mixtures. There was no association between antibody levels against any capture antigen and either clinical malaria incidence or parasite density.

**Conclusions:**

The current data shows that levels of naturally acquired antigen-specific antibodies, especially in infants and young children, are dependent on the antigenic allele used for measurement. This may be relevant to the interpretation of antibody titre data from measurements against single *Pf*AMA1 alleles, especially in studies involving infants and young children who have experienced fewer infections.

## Background

Antibodies have a demonstrably crucial role in protection against clinical malaria and the measurement of malaria-specific antibodies and their correlation with protection against disease/infection is essential in field as well as vaccine trial studies. Anti-malarial antibodies participate in such effector mechanisms as complement-mediated parasite clearance, red cell invasion inhibition, direct neutralization of parasites/toxins and antibody-mediated cellular inhibition/cytotoxicity [1-5].

Antibodies are naturally induced against a host of parasite antigens, and in vivo protection may generally be based on the cumulative/synergistic effect of relevant responses rather than responses to any single antigen. Additionally, at the peak of an infection, high levels of the relevant antibodies, rather than their generation from memory may be necessary for protection [6,7]. The precise determination of anti-malarial antibody levels in field and vaccine studies in disease-endemic areas is therefore very crucial to data interpretation as well as for identifying antigen correlates of protection. Association of antibody levels with clinical protection from malaria may be complicated by the effects of previous antigen exposure and by the fact that some induced antibodies are mere surrogates of an induced response with no protective value [8,9].

For polymorphic parasite antigens, antibodies against one allelic form have been shown to react less with other alleles as a significant proportion of antibodies are directed against strain-specific epitopes, and this represents yet another limitation in antibody titre estimation. *Plasmodium falciparum *apical membrane antigen 1 (*Pf*AMA1), a type 1 integral membrane protein expressed in the merozoite and sporozoite stages of the parasite and a leading candidate for the development of a blood stage vaccine is one such antigen [10-17]. Polymorphism in *Pf*AMA1 is due to a number of non-random point mutations that occur in the antigen's ectodomain, an effect that has been associated with host immune pressure on the parasite [18,19]. Thus for a highly polymorphic antigen like apical membrane antigen 1 (AMA1), many variants of which are likely to be present in a single population, estimation of the true antibody levels can be challenging as antibody levels measured against any single *Pf*AMA1 allele may underestimate the true levels of persisting antibodies. This hypothesis was tested by comparing the anti-*Pf*AMA1 antibody levels in plasma samples collected prior to the low transmission season in a naturally exposed population against four single *Pf*AMA1 alleles and three different *Pf*AMA1 allele mixtures. The antigen mixtures are expected to have a variety of unique epitopes that would enhance binding of the broad spectrum of polyclonal anti-AMA1 antibodies in naturally exposed individuals. The study further assesses the association of antibody levels with the incidence of clinical malaria during the low transmission season as well as with previous exposure to parasites.

## Methods

### Ethics statement

The current study used archived human samples from a longitudinal cohort study conducted during the malaria seasons of 1994 and 1995. The original study was approved by the Ministry of Health in Ghana and ethical clearance was sought from the ethics committee of the Ministry of Health. Written informed consent was obtained from parents of participating children for the original study, but sample analyses in the current study were done anonymously.

### Study population and sampling

A random sample of 95 archived plasma samples drawn from the previous longitudinal cohort study (conducted at Dodowa, an area in Southern Ghana with seasonal transmission of mainly *P. falciparum*) was used in this study. A detailed description of the study site and sampling procedures has previously been published [20,21]. Malaria transmission in the study area was perennial, but was highest during the rainy season (May - November) and lowest during the dry season (December - April). The original study involved a total of 300 children between the ages of 3 and 15 years. Participants were actively followed up every week for the entire duration of the study (16 months) and clinical and parasitological data were collected at each visit. Blood samples were drawn from study participants at the beginning of the high transmission season (April 1994) as well as at the end of the season, prior to the beginning of the low transmission season (November 1994). Plasma samples used here were prepared from blood samples taken before the low transmission season in November 1994. Clinical malaria was defined as having a fever and/or an axillary temperature above 37.5°C, as well as parasitaemia above 5000 parasites/μl of blood.

### Antibody determination

Anti-*Pf*AMA1 antibodies titres in plasma samples were measured using an antigen capture ELISA. Plates were coated separately with 1 μg/ml of AMA1 alleles from the FVO (GenBank accession number AJ277646), HB3 (GenBank accession number U33277), 3D7 (GenBank accession number U65407) and CAMP (GenBank accession number M58545) parasite strains, as well as with 1 μg/ml of three different antigen mixtures; i) a mixture of three Diversity covering (DiCo) antigens whose design is based on the amino acid sequences of 355 naturally occurring *Pf*AMA1 alleles [22], ii) a mixture of the FVO, HB3, 3D7 and CAMP alleles, designated as *Four*, and iii) a mixture of all seven allelic antigens, designated as *Seven*. All antigen mixtures had equal microgram (μg) quantities of the component *Pf*AMA1 alleles. All antigens were expressed in *Pichia pastoris *and potential N-Glycosylation sites were removed by methodologies that have been previously described [14,22-24]. Plates were blocked with 200 μl/well of 3% BSA in PBS-Tween 20 (0.05%) for 1 h, after which 100 μl/well of plasma (diluted 1: 200 and titrated 3-fold over 8 duplicate wells) was added and incubated for 1 h. Bound antibodies were detected by incubation with 100 μl/well of 0.8 μg/ml alkaline phosphatase-conjugated goat anti-human IgG for 1 h. Colour development was with 1 mg/ml p-Nitrophenyl phosphate in DEA buffer (0.15% MgCl_2_.6H_2_O, 0.01% diethanolamine, pH = 9.8) for 30 min and optical density (OD) was measured at 405 nm. ODs were subsequently expressed in arbitrary units (AU) by the calibrator (hyperimmune human serum pool) included on each ELISA plate using the 4PL-based ADAMSEL programme (Remarque^®^), a data management system that is accessible from the EMVDA website http://www.malariaresearch.eu. One arbitrary unit (1 AU) is equivalent to the reciprocal plasma dilution that gives an OD of 1.0 over background.

### Statistical analyses

Antibody titres were log-transformed to achieve normality and stratified by age (3 to 5 years, 6 to 10 years and 11 to 15 years) in order to assess the effect of age/antigen exposure on the specificity of elicited antibodies. Antibody titres were compared for the same age group across capture antigens by one-way analysis of variance followed by Tukey Honest Significance Difference post hoc tests where necessary. The student *t *test was used to make pair-wise comparisons between titres of different age groups measured against the same capture antigen. Titres are also presented as boxplots per age group on each capture antigen. For each capture antigen, antibody titre variability amongst the different age groups was assessed by Levene's test for homogeneity of variances. The log-transformed titres against different capture antigen pairs were subsequently compared using Tukey mean-difference (TMD) or Bland-Altman plots [25], which assess the degree of agreement between same sample measurements by different methods (here different capture antigens). Since titres were log-transformed, the x-axis gives the geometric mean of the two antibody measurements for the sample against the capture antigen pair and the y-axis gives the ratio of titre measurements. Plots show a bold horizontal line (line of equality) indicating the geometric mean of titre differences (ideally at titre ratio = 1 or titre difference = 0) between the antigen pair and dotted lines indicate the 95% limits of agreement for the paired data distribution. The vertical axis has been modified to show fold difference instead of the absolute titre difference. Similar recognition of antibodies from the same individual by two different *Pf*AMA1 alleles would suggest a difference of zero (0) or a log (difference) of one (1). Thus the more distant data points are from the line of equality, the greater the binding preference of the same antibodies for one allele over the other.

Association between antibody levels against each of the capture antigens and cumulative incidence of clinical malaria (with the corresponding 95% confidence intervals) was estimated by the Kaplan-Meier method. Clinical malaria incidence rate estimation included all malaria episodes that met the case definition. Data from all 95 children sampled for this study were included in the analyses. Three parasite density categories (designated "1 < 760", "760 < 3000" and "3000+") were created based on cut-offs that ensured an equal distribution of samples, and these were compared with the non-parasitaemic group (designated "None"). The association between antibody levels (and age group) with previous exposure to infection (defined as categorical variables) were assessed separately for titres against each capture antigen using a linear regression model. For each antigen, Cox regression with a robust standard error was used to estimate the rate ratio and its 95% confidence interval. The exposure of interest, antibody levels, was modeled as a continuous variable transformed to log base 2 so that rate ratios indicate the decrease in incidence rate of malaria corresponding to a two-fold increase in antibody levels.

Analyses and plots were made using the R statistical package (version 2.13.0, 2011, R development core team) and STATA package (Statacorp, College Station, TX).

## Results

**Figure 1:**
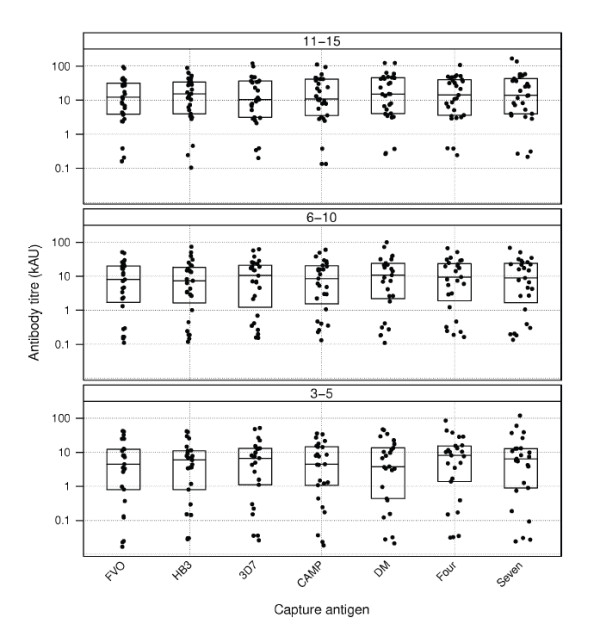
**Plasma antibody titres against *Pf*AMA1 alleles and allele mixtures**. Anti-*Pf*AMA1 antibody titres in plasma of malaria-exposed children were measured by an indirect ELISA using four *Pf*AMA1 alleles (from the FVO, HB3, 3D7 and CAMP parasite strains) and three different allele mixtures (DiCo mix or DM, a mixture of the four natural alleles, designated as *Four*, and a mixture of all seven alleles, designated as *Seven*) separately as capture antigens. Titres are presented for children aged 3 - 5 years (lower panel), 6 - 10 years (middle panel) and 11 - 15 years (upper panel). Each symbol represents plasma antibody titres of a study participant. Boxplots show the upper and lower quartiles as well as the median of each distribution. The vertical axis (antibody titre) is expressed in kilo arbitrary units (kAU), with one arbitrary unit being equivalent to the reciprocal plasma dilution that gives an OD of 1.0 over background.

The hypothesis that measurement of antibody levels in a malaria-endemic population against a polymorphic antigen would be influenced by the specific allele used was investigated in this study. Anti-*Pf*AMA1 antibody levels in the plasma of naturally exposed children was measured against four single *Pf*AMA1 alleles and compared with titres against three different *Pf*AMA1 allele mixtures (Figure 1). Antibody levels against all capture antigens/mixtures increased with age and mean levels against all antigens were significantly higher in the 11-15 year olds compared to the 3 - 5 year olds (*p *< 0.05, student *t *test). No significant differences were observed in antibody titres between the 6 - 10 year olds and the other two groups in separate comparisons (*p *> 0.05 in all cases, student *t *test). Antibody levels against different capture antigens/mixtures did not significantly differ for the same age groups (*P *> 0.05, one-way ANOVA).

**Figure 2:**
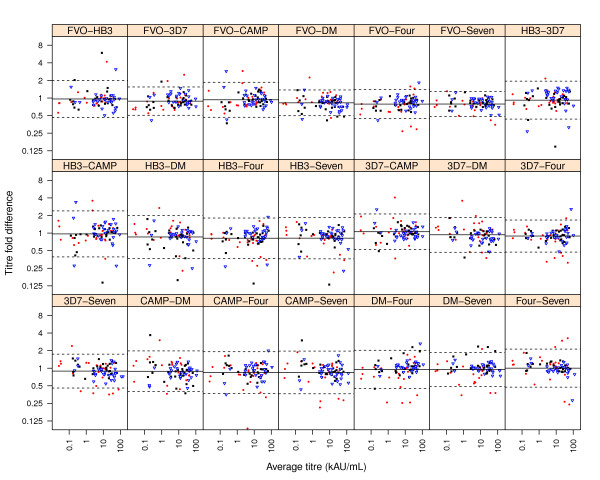
**Pair-wise comparison of anti-*Pf*AMA1 antibody titres against different capture antigens/mixtures**. Tukey Mean-Difference (TMD) plots were used to assess the level of agreement between antibody titres measured against pairs of capture antigens/mixtures. Each point represents a plot of the difference between two log-transformed titre measurements for a sample against the geometric mean of the same two measurements. For each panel, the bold horizontal line represents the average of all the differences between titres of the same samples against the indicated capture antigen/antigen mixture pair while the dotted horizontal lines represent the 95% limits of agreement for the distribution. Plot symbols represent individual data points; red filled circles are study participants aged 3 - 5 years, black filled squares are participants between 6 - 10 years and blue open triangles are participants between 11 - 15 years.

Results of the pair-wise comparison of antibody titres against the different capture antigens by TMD plots are presented in Figure 2. The more distant data points are from the line of equality, the greater the binding preference of the same antibodies for one allele over the other. On this premise, plot panels comparing antibody levels against single alleles indicate that younger children (3-5 years) showed a trend of greater strain-specificity, characterized by the slightly greater spread of data points (in red) compared to that of older children in most panels (Figure 2). This greater variability of antibody titres in younger children is also obvious from Figure 1 (Levene's test, *p *< 0.05 for all capture antigens except for the antigen mixtures *Four *and *Seven*).

Titre measurements of the polyclonal pool of antibodies in naturally exposed individuals would require an antigen(s) that present a broad range of antibody epitopes, a condition that may be fulfilled with multi-allele formulations. Three different allele mixtures (a mixture of the three DiCo antigens, designated DM, a mixture of the four single alleles, designated as *Four*, and a mixture of all seven *Pf*AMA1 alleles, designated as *Seven*) were used for titre measurements in this study. The best single alleles for antibody measurement in this population were determined by pair-wise comparison with titres against these allele mixtures. Antibody titres measured against the FVO and 3D7 alleles were least variable as judged by the narrow width of 95% limits of agreement (Figure 2) in separate pair-wise comparisons with all allele mixtures. Limits of agreement for the pair-wise comparison of titres against FVO and 3D7 alleles was also very narrow (Figure 2). The number of *Pf*AMA1 alleles (domains I, II and III) identified in four countries in the Africa region and whose amino acid sequences are similar to those of the four alleles used in this study are presented in Table 1. The sequences are part of published *Pf*AMA1 sequences that were retrieved from GenBank as of January 2011 (Remarque, personal communication). It is clear that all four alleles used in this study occur at very low frequencies in these populations (Table 1), though the 3D7 allele seems more prevalent compared to the FVO allele.

**Table 1:** Number of *Pf*AMA1 alleles with similar amino acid sequences to alleles used in this study.

*Pf*AMA1 allele		Country		
	
	Gambia	Mali	Nigeria	Kenya
FVO	1	0	0	0

HB3	0	1	0	0

3D7	2	23	2	1

CAMP	3	6	1	1

*Number of sequences	127	923	52	143

*This is the total number of *Pf*AMA1 amino acid sequences from those countries based on published data as of January 2011.

The current study also investigated the association of antibody levels with the cumulative incidence of clinical malaria as well as with previous exposure to *Plasmodium *parasites. Of the 95 children whose samples and clinical data were available for analysis, 23 had at least one clinical episode during the low transmission season and the incidence rate of malaria decreased with the age of participants (Table 2). There was however no association between antibody levels against any of the capture antigens and the incidence of clinical malaria before and after correction for age (Table 3).

**Table 2:** Characteristics of study population

Characteristics	Number of children (%)	Cumulative incidence (95%CI)	Child-months at risk	Malaria cases	Rate per 100 child-months (95% CI)
**Age group**					

3 - 5 years	39 (41)	20.1% (10, 38.1)	318.82	11	3.5 (1.9, 6.2)

6 - 10 years	27 (28)	22.4% (10.7, 43.2)	226.63	7	3.1 (1.5, 6.5)

11 - 15 years	29 (31)	17.6% (7.7, 37.3)	243.58	5	2.1 (0.9, 4.9)

**TOTAL**	95	19.8% (13, 29.7)	789.03	23	2.9 (1.9, 4.4)

Clinical malaria is defined as a history of fever or temperature > = 37.5°C and parasite density > = 5000/μl

**Table 3:** Age-adjusted incidence rate ratio (IRR) for the association of anti-parasite antibody levels with malaria incidence

Antibody	Antigen	Crude HR (95%CI)	HR adjusted for age (95%CI)	*P*-value for adjusted HR
IgG	AMA1-FVO	1.06 (0.92, 1.22)	1.09 (0.93,1.27)	0.3

	AMA1-3D7	1.05 (0.92, 1.19)	1.08 (0.94, 1.24)	0.3

	AMA1-DM	1.05 (0.92, 1.21)	1.08 (0.93, 1.26)	0.32

	AMA1-FOUR	1.07 (0.93, 1.23)	1.09 (0.94, 1.27)	0.25

	AMA1-SEVEN	1.06 (0.92, 1.21)	1.08 (0.93, 1.26)	0.3

	AMA1-CAMP	1.08 (0.94, 1.24)	1.11 (0.95, 1.30)	0.19

	AMA1-HB3	1.04 (0.91, 1.18)	1.07 (0.92,1.24)	0.39

**Figure 3:**
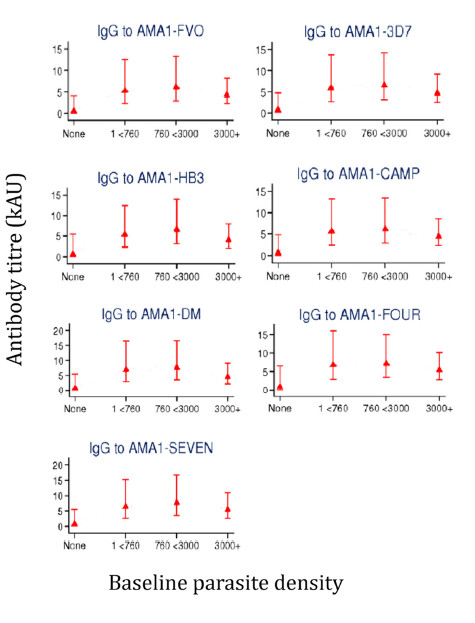
**Geometric mean of antibody titres for study participants with different parasite densities**. Error bars show the 95% CI of antibody titres per parasite density category. Sample size; n = 10 for the group "None", n = 29 for "1 < 760", n = 28 for "760 < 3000" and n = 28 for the group "3000+" in all panels.

Association of anti-AMA1 titres against the different capture antigens with previous exposure to parasites within the preceding 6 months of the study was investigated using linear regression models. Participants were grouped by their geometric mean parasite density into four categories and the relationship with antibody levels against each capture antigen assessed. Participants who had no detectable parasites (None, n = 10) had the lowest anti-AMA1 antibody levels (Figure 3). Participants in this group had had no infection during the preceding 6 months before the plasma samples analyzed here were taken, but might have experienced infections prior to the start of the study. Participants who had experienced moderate parasite densities (two groups, 1 < 760 and 760 < 3000 parasites/μl of blood, n = 29 and n = 28, respectively) had the highest levels of antibodies when compared with those of participants with no exposure within the preceding 6 months. Participants who had previously experienced parasite densities greater than 3000/μl of blood (3000*+*, n = 28, Figure 3) however had antibody levels intermediate between those of the unexposed (*None*) and the other two exposed groups (1 < 760 and 760 < 3000). Despite these trends, there were no significant differences in antibody levels between any two parasite density groups. However, of the 23 children who had at least one clinical episode, 18 were in the "3000+" parasite density category and the remaining 5 were in the "1 < 760" category.

## Discussion

An accurate estimation of antibody levels against malaria parasite antigens is necessary for establishing antibody correlates of protection against malaria in human field studies and clinical trials. A number of parasite antigens that are currently being assessed as vaccine candidates show polymorphism, and the estimation of antibody titres using a single allelic form may be confounded by the occurrence of a large number of allelic variants in nature.

In this study, the effect of antigenic polymorphism on the measured levels of anti-*Pf*AMA1 antibodies was assessed by comparing titres against single *Pf*AMA1 alleles and allele mixtures in plasma samples taken before the low malaria transmission season in a previous study. The *Pf*AMA1 allele mixtures are expected to present a broad spectrum of antigenic epitopes that will be recognised by antibodies in the polyclonal pool. We also investigated the association of anti-*Pf*AMA1 antibody levels with the cumulative incidence of clinical malaria as well as with previous exposure to parasites.

For participants in any one age group, antibody levels against the different capture antigens/mixtures did not differ significantly. This observation is not surprising as antibodies in the plasma samples from most study participants were most likely induced after repeated infection with diverse parasite strains over time. Several studies have indeed reported a large diversity of parasite strains within single communities in disease-endemic areas [19,26-28]. Genotyping of parasite strains in study participants here would have added to data interpretation, but this could not be done due to the unavailability of matching DNA samples.

The greater variability of antibody titres (against all but the antigen mixtures *Four *and *Seven*) observed in younger children (Figures 1 and 2) suggests that individuals with fewer parasite exposures might have a greater proportion of strain-specific antibodies compared to individuals who have had many parasite infections. It is therefore possible that the use of a single allele for antibody measurement, especially in infants/young children or individuals with limited parasite exposure, may under-estimate antibody levels and such data need to be interpreted cautiously.

The data here also suggest that though humans discriminate between *Pf*AMA1 alleles and possibly other polymorphic parasite antigens, these effects may become less apparent with age and exposure to variant parasite strains. This observation is in agreement with previous observations in humans [29]. In a study involving participants from Papua New Guinea for example, Cortes et al. [30] showed that the majority of anti-*Pf*AMA1 antibodies directed against polymorphic epitopes were detected in younger age groups compared to older individuals. The development of a more cross-reactive profile with age/exposure may most likely be as a result of clonal imprinting, with antigens derived from every infection primarily boosting memory to previously encountered antigenic epitopes. This phenomenon has been demonstrated in a controlled setting in rabbits after immunisation with different *Pf*AMA1 alleles in sequence [31].

Antibody measurement against a *Pf*AMA1 allele mixture is expected to give the best titre estimate of polyclonal anti-*Pf*AMA1 antibodies in the field since the mixture would have a large diversity of antigenic epitopes. Such an approach based on a practical number of alleles may be useful, especially since there is a high likelihood of many parasite strains occurring in a given community, and even when there are a limited number of strains, their specific AMA1 alleles may not be readily available for use as coating antigens.

Three different allele mixtures were used for titre measurements in this study. DiCo mix (DM), one of the mixtures, consists of three DiCo antigens that were designed based on the sequences of 355 naturally occurring *Pf*AMA1 alleles to inherently cover naturally occurring polymorphism in *Pf*AMA1 [22]. The mixture has been shown in our laboratory to yield higher antibody titres in samples from naturally exposed individuals compared to titres against single natural alleles (unpublished data). Titres against the FVO and 3D7 AMA1 alleles were most comparable to titres against DM and the other two allele mixtures, suggesting that the FVO and 3D7 alleles preferentially recognize a greater number of antibody specificities compared to the CAMP and HB3 alleles. This is against the observation that all four single alleles used in this study have only been found at very low frequencies in the Africa region (Table 1), though the 3D7 allele seems more prevalent compared to the FVO allele. Thus the measured levels of circulating antibodies in a population with multiple strain infections are dependent on the choice of *Pf*AMA1 alleles used for titre measurement.

The low prevalence of all four alleles shows the complexity involved in the measurement and evaluation of responses to *Pf*AMA1 in a naturally exposed population as even in the current study there may still be strain-specific anti-*Pf*AMA1 antibodies that may not be detected by the best single alleles. It is however worth mentioning that the levels of naturally induced cross-reactive antibodies seem to be the repertoire required for cross protection [31-33], and current vaccine strategies aim at inducing such cross-strain antibodies. Antibody measurement against a single allele could therefore give an indication of the levels of cross-strain (protective) antibodies.

There was no association between antibody levels against any of the capture antigens and either the incidence of clinical malaria or previous exposure to parasites before and after correction for age. Antibody levels against *Pf*AMA1 have been associated with a reduced risk of malaria incidence in other studies with comparable population characteristics [34-37]. While the association of antibody levels with clinical protection in these studies was assessed during the high transmission season, association in the current study was assessed during the low transmission season. An earlier study in the same population measured anti-MSP1_19 _antibodies and reported similar levels of antibodies at the beginning and end of the high transmission season [20]. This study also showed no correlation between anti-MSP1_19 _antibodies and protection from disease. These aside, the limited sample size in the current study is also likely to reduce the statistical power required to detect significant associations.

Finally, the in vitro functionality of antibodies could not be assessed as the amount of plasma available was very limited and assays would need to be performed with multiple strains in order to assess the specificity of functional antibodies.

There was a trend of decreasing antibody levels with increasing parasite density from previous exposure, but this inverse association was not statistically significant. This notwithstanding, the observation that 18 of the 23 children who experienced clinical malaria during the study period were in the high parasitaemia group (3000+) suggests that children with moderate to low parasitaemia (below 3000 parasites/μl of blood) were less susceptible to disease. This is in agreement with the observation that maintenance of immune effectors involved in conferring protection against disease requires the persistence of low levels of circulating parasites [29,38].

## Conclusions

The data presented generally suggests a cautious selection of antigens for the measurement of naturally induced antibody levels against polymorphic targets, especially in samples from individuals with limited exposure to parasites. Though the study utilized archived samples taken during the 1994/1995 malaria season, the findings have direct relevance for the assessment of naturally acquired antibodies of broad specificity since titres are measured against randomly selected (allelic) antigens and the exact circulating strains/antigenic alleles within study populations are usually not taken into account. Alternatively, DiCo mix may represent an ideal candidate for the measurement of antibody titres in naturally exposed populations, especially in infants and young children as the three DiCo antigens, apart from the effect of mixing, have been designed to cover polymorphism that is seen in naturally occurring *Pf*AMA1 alleles. The data also points to a trend of increasing proportion of antibodies against cross-strain epitopes with age, and suggest the involvement of clonal imprinting in the development of this antibody repertoire. Finally, though there was no association of antibody levels with either a reduced incidence of clinical malaria or previous exposure to parasites, individuals with moderate parasitaemia (< 3000/μl) had higher absolute antibody levels than those with parasitaemia greater than 3000/μl of blood. These findings are collectively relevant to the interpretation of data on antibodies against polymorphic antigens, especially in field studies involving groups with limited parasite exposure.

## Competing interests

We have read the journal's policy and have the following conflicts; Three of the authors are in the process of obtaining a patent for the three synthetic Diversity-Covering (DiCo) AMA1 proteins. This does not alter their adherence to all *BMC Infectious Diseases *policies on sharing data and materials. All other authors have no competing interests.

## Authors' contributions

Conceived and designed the experiments: EJR KAK BWF DD. Performed the experiments: KAK. Analyzed the data: KAK EJR SB. Designed and produced recombinant proteins: BWF, MvdE. Wrote the paper: KAK EJR BWF CHMK DD. All authors have read and approved the final manuscript.

## Pre-publication history

The pre-publication history for this paper can be accessed here:

http://www.biomedcentral.com/1471-2334/12/32/prepub

## References

[B1] Blackman MJ Scott-Finnigan TJ Shai S Holder AA Antibodies inhibit the protease-mediated processing of a malaria merozoite surface protein J Exp Med 1994 180 389 393 10.1084/jem.180.1.389 7516416 PMC2191569

[B2] Deans JA Alderson T Thomas AW Mitchell GH Lennox ES Cohen S Rat monoclonal antibodies which inhibit the *in vitr *multiplication of *Plasmodium knowles* Clin Exp Immunol 1982 49 297 309 6751636 PMC1536485

[B3] Dutta S Haynes JD Moch JK Barbosa A Lanar DE Invasion-inhibitory antibodies inhibit proteolytic processing of apical membrane antigen 1 of *Plasmodium falciparum *merozoites Proc Natl Acad Sci USA 2003 100 12295 12300 10.1073/pnas.2032858100 14526103 PMC218752

[B4] Schofield L Hewitt MC Evans K Siomos MA Seeberger PH Synthetic GPI as a candidate anti-toxic vaccine in a model of malaria Nature 2002 418 785 789 10.1038/nature00937 12181569

[B5] Theisen M Soe S Oeuvray C Thomas AW Vuust J Danielsen S Jepsen S Druilhe P The glutamate-rich protein (GLURP) of *Plasmodium falciparum *is a target for antibody-dependent monocyte-mediated inhibition of parasite growth *in vitr* Infect Immun 1998 66 11 17 9423833 10.1128/iai.66.1.11-17.1998 PMC107852

[B6] Lambert PH Liu M Siegrist CA Can successful vaccines teach us how to induce efficient protective immune responses? Nat Med 2005 11 S54 S62 10.1038/nm1216 15812491

[B7] Struik SS Riley EM Does malaria suffer from lack of memory? Immunol Rev 2004 201 268 290 10.1111/j.0105-2896.2004.00181.x 15361247

[B8] Bousema T Drakeley C Gesase S Hashim R Magesa S Mosha F Otieno S Carneiro I Cox J Msuya E Kleinschmidt I Maxwell C Greenwood B Riley E Sauerwein R Chandramohan D Gosling R Identification of hot spots of malaria transmission for targeted malaria control J Infect Dis 2010 201 1764 1774 10.1086/652456 20415536

[B9] Greenhouse B Ho B Hubbard A Njama-Meya D Narum DL Lanar DE Dutta S Rosenthal PJ Dorsey G John CC Antibodies to *Plasmodium falciparum *antigens predict a higher risk of malaria but protection from symptoms once parasitemic J Infect Dis 2011 204 19 26 10.1093/infdis/jir223 21628654 PMC3105040

[B10] Crewther PE Matthew ML Flegg RH Anders RF Protective immune responses to apical membrane antigen 1 of *Plasmodium chabaud *involve recognition of strain-specific epitopes Infect Immun 1996 64 3310 3317 8757869 10.1128/iai.64.8.3310-3317.1996 PMC174223

[B11] Dutta S Lee SY Batchelor AH Lanar DE Structural basis of antigenic escape of a malaria vaccine candidate Proc Natl Acad Sci USA 2007 104 12488 12493 10.1073/pnas.0701464104 17636123 PMC1941496

[B12] Hodder AN Crewther PE Anders RF Specificity of the protective antibody response to apical membrane antigen 1 Infect Immun 2001 69 3286 3294 10.1128/IAI.69.5.3286-3294.2001 11292751 PMC98287

[B13] Kennedy MC Wang J Zhang Y Miles AP Chitsaz F Saul A Long CA Miller LH Stowers AW *In vitr *studies with recombinant *Plasmodium falciparum *apical membrane antigen 1 (AMA1): production and activity of an AMA1 vaccine and generation of a multiallelic response Infect Immun 2002 70 6948 6960 10.1128/IAI.70.12.6948-6960.2002 12438374 PMC133034

[B14] Kusi KA Faber BW Thomas AW Remarque EJ Humoral immune response to mixed *P*AMA1 alleles; multivalent *P*AMA1 vaccines induce broad specificity PLoS ONE 2009 4 e8110 10.1371/journal.pone.0008110 19956619 PMC2779588

[B15] Silvie O Franetich JF Charrin S Mueller MS Siau A Bodescot M Rubinstein E Hannoun L Charoenvit Y Kocken CH Thomas AW Geert-Jan van Gemert GJ, Sauerwein RW, Blackman MJ, Anders RF, Pluschke G, Mazier D: A role for apical membrane antigen 1 during invasion of hepatocytes by Plasmodium falciparum sporozoites J Biol Chem 2004 279 9490 9496 10.1074/jbc.M311331200 14676185

[B16] Stowers AW Kennedy MC Keegan BP Saul A Long CA Miller LH Vaccination of monkeys with recombinant *Plasmodium falciparum *apical membrane antigen 1 confers protection against blood-stage malaria Infect Immun 2002 70 6961 6967 10.1128/IAI.70.12.6961-6967.2002 12438375 PMC133036

[B17] Thomas AW Waters AP Carr D Analysis of variation in PF83, an erythrocytic merozoite vaccine candidate antigen of *Plasmodium falciparum* Mol Biochem Parasitol 1990 42 285 287 10.1016/0166-6851(90)90172-I 2270110

[B18] Escalante AA Lal AA Ayala FJ Genetic polymorphism and natural selection in the malaria parasite *Plasmodium falciparum* Genetics 1998 149 189 202 9584096 10.1093/genetics/149.1.189 PMC1460124

[B19] Polley SD Chokejindachai W Conway DJ Allele frequency-based analyses robustly map sequence sites under balancing selection in a malaria vaccine candidate antigen Genetics 2003 165 555 561 14573469 10.1093/genetics/165.2.555 PMC1462796

[B20] Dodoo D Theander TG Kurtzhals JA Koram K Riley E Akanmori BD Nkrumah FK Hviid L Levels of antibody to conserved parts of *Plasmodium falciparum *merozoite surface protein 1 in Ghanaian children are not associated with protection from clinical malaria Infect Immun 1999 67 2131 2137 10225865 10.1128/iai.67.5.2131-2137.1999 PMC115948

[B21] Dodoo D Staalsoe T Giha H Kurtzhals JA Akanmori BD Koram K Dunyo S Nkrumah FK Hviid L Theander TG Antibodies to variant antigens on the surfaces of infected erythrocytes are associated with protection from malaria in Ghanaian children Infect Immun 2001 69 3713 3718 10.1128/IAI.69.6.3713-3718.2001 11349035 PMC98376

[B22] Remarque EJ Faber BW Kocken CH Thomas AW A diversity-covering approach to immunization with *Plasmodium falciparum *apical membrane antigen 1 induces broader allelic recognition and growth inhibition responses in rabbits Infect Immun 2008 76 2660 2670 10.1128/IAI.00170-08 18378635 PMC2423090

[B23] Faber BW Remarque EJ Kocken CH Cheront P Cingolani D Xhonneux F Jurado M Haumont M Jepsen S Leroy O Thomas AW Production, quality control, stability and pharmacotoxicity of cGMP-produced *Plasmodium falciparum *AMA1 FVO strain ectodomain expressed in *Pichia pastori* Vaccine 2008 26 6143 6150 10.1016/j.vaccine.2008.08.055 18804135

[B24] Kocken CH Withers-Martinez C Dubbeld MA van der WA Hackett F Valderrama A Blackman MJ Thomas AW High-level expression of the malaria blood-stage vaccine candidate Plasmodium falciparum apical membrane antigen 1 and induction of antibodies that inhibit erythrocyte invasion Infect Immun 2002 70 4471 4476 10.1128/IAI.70.8.4471-4476.2002 12117958 PMC128198

[B25] Bland JM Altman DG Agreement between methods of measurement with multiple observations per individual J Biopharm Stat 2007 17 571 582 10.1080/10543400701329422 17613642

[B26] Osier FH Weedall GD Verra F Murungi L Tetteh KK Bull P Faber BW Remarque E Thomas A Marsh K Conway DJ Allelic diversity and naturally acquired allele-specific antibody responses to *Plasmodium falciparum *apical membrane antigen 1 in Kenya Infect Immun 2010 78 4625 4633 10.1128/IAI.00576-10 20732997 PMC2976345

[B27] Polley SD Conway DJ Strong diversifying selection on domains of the *Plasmodium falciparum *apical membrane antigen 1 gene Genetics 2001 158 1505 1512 11514442 10.1093/genetics/158.4.1505 PMC1461755

[B28] Takala SL Coulibaly D Thera MA Batchelor AH Cummings MP Escalante AA Ouattara A Traore K Niangaly A Djimde AA Doumbo OK Plowe CV Extreme polymorphism in a vaccine antigen and risk of clinical malaria: implications for vaccine development Sci Transl Med 2009 1 2 5 10.1126/scitranslmed.3000257 PMC2822345 20165550

[B29] Doolan DL Dobano C Baird JK Acquired immunity to malaria Clinical Microbiology Reviews 2009 22 13 36 10.1128/CMR.00025-08 19136431 PMC2620631

[B30] Cortes A Mellombo M Masciantonio R Murphy VJ Reeder JC Anders RF Allele specificity of naturally acquired antibody responses against *Plasmodium falciparum *apical membrane antigen 1 Infect Immun 2005 73 422 430 10.1128/IAI.73.1.422-430.2005 15618180 PMC538974

[B31] Kusi KA Faber BW van der EM Thomas AW Kocken CH Remarque EJ Immunization with different *P*AMA1 alleles in sequence induces clonal imprint humoral responses that are similar to responses induced by the same alleles as a vaccine cocktail in rabbits Malar J 2011 10 40 10.1186/1475-2875-10-40 21320299 PMC3050776

[B32] Dutta S Sullivan JS Grady KK Haynes JD Komisar J Batchelor AH Soisson L Diggs CL Heppner DG Lanar DE Collins WE Barnwell JW High antibody titer against apical membrane antigen-1 is required to protect against malaria in the *Aotu *model PLoS ONE 2009 4 e8138 10.1371/journal.pone.0008138 19997632 PMC2780715

[B33] Kusi KA Faber BW Riasat V Thomas AW Kocken CH Remarque EJ Generation of humoral immune responses to multi-allele *P*AMA1 vaccines; effect of adjuvant and number of component alleles on the breadth of response PLoS ONE 2010 5 e15391 10.1371/journal.pone.0015391 21082025 PMC2972715

[B34] Dodoo D Aikins A Kusi KA Lamptey H Remarque E Milligan P Bosomprah S Chilengi R Osei YD Akanmori BD Theisen M Cohort study of the association of antibody levels to AMA1, MSP1_19_, MSP3 and GLURP with protection from clinical malaria in Ghanaian children Malar J 2008 7 142 10.1186/1475-2875-7-142 18664257 PMC2529305

[B35] Dodoo D Atuguba F Bosomprah S Ansah NA Ansah P Lamptey H Egyir B Oduro AR Gyan B Hodgson A Koram KA Antibody levels to multiple malaria vaccine candidate antigens in relation to clinical malaria episodes in children in the Kasena-Nankana district of Northern Ghana Malar J 2011 10 108 10.1186/1475-2875-10-108 21529376 PMC3101166

[B36] Fowkes FJ Richards JS Simpson JA Beeson JG The relationship between anti-merozoite antibodies and incidence of *Plasmodium falciparum *malaria: A systematic review and meta-analysis PLoS Med 2010 7 e1000218 10.1371/journal.pmed.1000218 20098724 PMC2808214

[B37] Iriemenam NC Khirelsied AH Nasr A Elghazali G Giha HA Elhassan A-E Agab-Aldour AA Montgomery SM Anders RF Theisen M Troye-Blomberg M Elbashir MI Berzins K Antibody responses to a panel of *Plasmodium falciparum *malaria blood-stage antigens in relation to clinical disease outcome in Sudan Vaccine 2009 27 62 71 10.1016/j.vaccine.2008.10.025 18977268

[B38] Gatton ML Cheng Q Investigating antigenic variation and other parasite-host interactions in *Plasmodium falciparum *infections in naive hosts Parasitology 2004 128 367 376 10.1017/S0031182003004608 15151141

